# The association between community environment and depression among Chinese older adults: an age-stratified analysis of the dual mediation pathways of aging anxiety and social adaptation stress

**DOI:** 10.3389/fpubh.2026.1783732

**Published:** 2026-04-22

**Authors:** Dongmei Wu, Zihong Chen, Zhiyuan Chi, Fanglong Wang

**Affiliations:** College of Art and Design, Jiangsu Ocean University, Lianyungang, Jiangsu, China

**Keywords:** age differences, aging anxiety, community environment, depression, older adults, social adaptation stress

## Abstract

**Introduction:**

With the accelerating pace of population aging in China, depression among older adults has emerged as a significant public health challenge. Existing research has yet to fully elucidate the underlying processes linking community environment to depression among older adults, particularly from an age-differentiated perspective.

**Methods:**

To address this gap, this study constructed an integrated model positing aging anxiety and social adaptation stress as mediators and age as a moderator, aiming to systematically examine the pathways associated with the relationship between community environment and depression among older adults and their heterogeneity across age groups. Using valid data from 10,562 respondents in the 2023 China Longitudinal Aging Social Survey (CLASS 2023), the analysis was conducted via structural equation modeling.

**Results:**

The findings indicate: first, a significant negative correlation exists between community environment and depression; second, both aging anxiety and social adaptation stress exhibit significant indirect associations in this relationship, with the indirect effect of social adaptation stress being stronger. More importantly, the association between community environment and depression is stronger for older-old adults and is primarily linked to the pathway involving aging anxiety. In contrast, its association with depression in younger-old adults is channeled to a larger extent through social adaptation stress.

**Discussion:**

This study unveils the pattern of dual pathways and differential patterns related to age that characterize the relationship between community environment and depression among older adults. The findings provide empirical evidence and theoretical support for future efforts to build age friendly communities and implement stratified healthy aging policies.

## 1 Introduction

China has witnessed a marked acceleration in population aging, with the number of older adults increasing steadily year by year. According to the National Bureau of Statistics, by the end of 2024, the population aged 60 and above had surpassed 280 million, accounting for 19.8% of the total population (1). Within this demographic transition, the health status of older adults has emerged as a focal point of societal and academic concern. As physiological function inevitably declines with age, the risk of mental health problems among older adults escalates proportionally. The Healthy Aging Development Report indicates that approximately 18.6% of older adults currently exhibit depressive tendencies, highlighting depression as a significant public health issue affecting quality of life in this population (2). Research on depression in older adults thus carries substantial practical significance–not only for individual health but also for alleviating caregiving burdens on families and society and for advancing the broader goals of healthy aging (3). While previous studies have often addressed this issue from the perspective of factors at the individual level or family dynamics (4, 5), the role of the community environment as a key determinant of depression in later life remains relatively underexplored.

As primary users of community environments, the vast majority of older adults in China concentrate their daytime activities within and around their neighborhoods. Their daily routines–including travel, social interaction, and leisure pursuits–are profoundly shaped by the quality of their immediate surroundings. Consequently, in recent years, a growing body of scholarship has turned its attention to the relationship between community environments and the health of older adults. Studies have documented that community environmental factors, including green space accessibility, walkability, and perceived safety, are significantly associated with the mental health of older persons (6–9). Additionally, community social capital and neighborhood cohesion have been linked to fewer depressive symptoms, appearing to buffer psychological distress by providing emotional support and reducing social adaptation stress (10, 11). However, most of this evidence comes from geographically limited surveys carried out in specific urban or rural areas, leaving a gap in research that covers the full national context at a broad scale.

As research in environmental health advances, a growing number of scholars recognize that the relationship between community environment and depression among older adults is not simply linear but involves complex mechanistic pathways. For example, the accessibility and quality of community green spaces are significantly associated with a lower risk of depression, with underlying mechanisms including the provision of recreational venues and the promotion of physical activity (12–14). Furthermore, high-quality built environments have been shown to encourage older adults to engage in outdoor activities, thereby reducing social adaptation stress and enhancing mental health (15, 16). Most existing mediation models are grounded in the “environment–behavior–health” framework derived from socioecological theory (17). While physical activity serves as a key behavioral bridge connecting environment and health in older adults, their social perception also plays a mediating role to a certain extent (18, 19). Against the backdrop of rapidly aging societies, this study further incorporates social adaptation theory to provide a more nuanced explanation of the mechanisms through which community environment shapes the mental health of older adults. Social adaptation theory posits that an individual's capacity to maintain stable cognitive, emotional, and behavioral functioning in response to environmental changes, societal transitions, and role adjustments is pivotal to their mental health. For older adults, the degree to which they adapt to social change, community contexts, and normative expectations is closely tied to their levels of psychological distress, social alienation, and overall mental health status (20). The selection of social adaptation stress and aging anxiety as mediators is well justified within the framework of social adaptation theory. Social adaptation stress represents a state of objective breakdown in social connections in the present, serving as a direct social consequence of adverse environmental influences (21). Aging anxiety, by contrast, reflects a fear oriented toward the future regarding potential breakdowns in social networks, and constitutes an anticipatory psychological threat triggered by unfavorable environmental conditions. Together, they form a comprehensive psychosocial pathway linking community environment to health outcomes (22). However, few existing studies have treated the social perception of older adults as a mediator, and even fewer have simultaneously incorporated both aging anxiety and social adaptation stress into their investigations.

This study constructs a theoretical framework in which social adaptation stress and aging anxiety are designated as parallel mediating variables. This deliberate choice is grounded in their conceptual distinctiveness and the independent pathways through which each links community environment to depression. Specifically, drawing on social adaptation theory, social adaptation stress refers to the psychological tension and strain experienced by older adults when their adaptive capacity falls short of environmental demands in the context of community changes and normative adjustments. It is an affective and psychological variable centered on the state of maladaptation between the individual and their community environment. Aging anxiety, by contrast, embodies fears and concerns regarding physical decline and diminished social worth, representing a psychological and cognitive variable focused on subjective appraisals and emotional responses to the aging process itself. Moreover, informed by social adaptation theory, the community environment can simultaneously activate these two relatively independent pathways. On the one hand, communities with lower levels of age-friendliness may increase the adaptive burden on older adults, heighten their social adaptation stress, and consequently contribute to depressive symptoms. On the other hand, a disadvantaged environment may also serve as an implicit cue of aging, reinforcing negative self-perceptions of growing old and directly triggering aging anxiety, which in turn undermines mental health. Although social adaptation stress and aging anxiety may be interrelated, imposing a serial mediation model would imply a strong causal assumption that objective adaptive stress unidirectionally determines subjective cognitive and emotional states. Such a model would not only overlook older adults who experience aging anxiety due to environmental cues without manifesting significant social adaptation stress, but also risk underestimating the true association between community environment and mental health through independent cognitive pathways. Thus, incorporating social adaptation stress and aging anxiety as parallel mediators enables a more comprehensive and ecologically valid portrayal of the multifaceted psychosocial mechanisms through which community environment influences depression in later life. This approach aligns with the core tenet of social adaptation theory–namely, the dynamic interplay between individual and environment in achieving adaptive fit–while avoiding the theoretical oversimplification inherent in presuming a unidirectional chain of effects. It better reflects the complex, dynamic, and concurrent psychological adaptation processes characteristic of older adults' lived experiences.

Furthermore, older adulthood, a life stage spanning several decades, is characterized by considerable heterogeneity within this population. Developmental psychology and gerontology have long recognized that older adults of different ages exhibit systematic differences in physical function, social roles, and psychological needs (23). In terms of mental health, younger-old adults may face greater psychological adjustment pressures arising from social role transitions such as retirement, while generally retaining a stronger willingness and capacity for social engagement. By contrast, older-old adults are more likely to experience pronounced social adaptation stress and psychological vulnerability due to physical limitations, chronic illness, and the erosion of peer networks (24, 25). This divergence, rooted in distinct life stages, suggests that the mechanisms through which community environments relate to psychosocial processes may differ in strength or pathway across age groups (26). Against this backdrop, to more fully explore the boundary conditions of these associations, the present study incorporates age as a key moderating variable within the analytical framework, aiming to empirically test whether the links between community environment and social adaptation stress, aging anxiety, and depression exhibit differential patterns across age subgroups.

Based on the analysis above, this study draws on data from the 2023 China Longitudinal Aging Social Survey (CLASS 2023) and employs structural equation modeling to construct and test a conceptual framework linking community environment to depression among older adults, with aging anxiety and social adaptation stress as parallel mediators. It further examines the moderating role of age in these associations. The contributions are twofold. Theoretically, by integrating social adaptation theory, the study uncovers a dual pathway mechanism through which community environment relates to depressive symptoms. The examination of age-related heterogeneity further reveals how these pathways operate differently across subgroups of older adults, offering a life course perspective on environmental health research. Practically, the findings provide actionable insights for age-friendly community development. They underscore the need for environmental interventions that simultaneously address physical infrastructure and social connectedness, while also advocating for age-differentiated strategies tailored to the distinct needs of younger-old and older-old populations. These insights offer empirically grounded support for the implementation of healthy aging policies.

## 2 Materials and methods

### 2.1 Data and samples

The China Longitudinal Aging Social Survey (CLASS) is a nationally representative, large scale longitudinal survey project. It aims to comprehensively capture the personal health and socioeconomic circumstances of older adults in China, monitor their support resources, attitudes toward aging, and care needs, dynamically trace the physiological, psychological, and social aging processes of the country's older population, and provide an empirical foundation for the formulation of scientifically grounded aging policies. The present study draws on data from the CLASS 2023 follow up survey. This wave of data collection employed a multistage stratified probability sampling design, covering 30 provinces, autonomous regions, and municipalities directly under the central government, across 462 neighborhood and village committees. The sampling procedure proceeded as follows: first, administrative villages and communities were designated as primary sampling units, with sample sites randomly selected based on a map address frame; subsequently, households were randomly drawn from within these selected sites; finally, within each sampled household, one adult aged 60 or above was randomly selected using the Kish grid method for face-to-face interview, yielding a total of 11,670 valid responses.

Prior to the formal analysis, we examined the pattern of missing data. The original sample consisted of 11,670 respondents, of whom 1,108 (approximately 9.5%) had item non-response on core study variables. To assess the missingness mechanism, we conducted logistic regression analyses, which revealed that missingness was associated with certain demographic characteristics (e.g., age, education level), suggesting that the data were not missing completely at random (MCAR) but rather conformed to the missing at random (MAR) assumption. Given the relatively large sample size and the fact that 10,562 valid cases remained after excluding missing observations—ensuring sufficient statistical power—we employed listwise deletion as the primary analytic approach. Under MAR conditions, listwise deletion yields consistent and efficient parameter estimates and exhibits considerable robustness in large samples. To further evaluate the potential impact of the missing data handling method on our conclusions, we conducted a sensitivity analysis using multiple imputation (MI). The imputation model included all analytic variables and key sociodemographic covariates. Following Rubin's (1987) rules, we generated 20 complete datasets to reduce sampling variation, re-estimated the structural equation models on the imputed datasets, and compared the pooled estimates with those obtained from listwise deletion. Additionally, following the age classification standards of the World Health Organization, the entire valid sample was divided into two groups: younger-old adults (aged 60–74) and older-old adults (aged 75 and above). This resulted in a younger-old group of 6,622 individuals and an older-old group of 3,940 individuals, facilitating subsequent subgroup comparison and analysis.

### 2.2 Sample characteristics

Table 1 presents the characteristics of the sample included in this study. For categorical variables, frequencies and percentages are reported; for continuous variables, means are reported. In terms of sociodemographic characteristics, the mean age of older adults in the sample was 73.321 years. The gender distribution was balanced, with males accounting for 51.9% and females for 48.1%. No significant difference in gender composition was observed between the younger-old and older-old groups. Regarding educational attainment, the sample's education level was primarily concentrated at the elementary and middle school levels, reflecting the generally low educational attainment characteristic of the older adult population. The educational level of the younger-old group was significantly higher than that of the older-old group, with only 12.0% of the younger-old being illiterate–considerably lower than the 28.1% in the older-old group. This reflects intergenerational differences in educational access associated with societal development. In terms of marital status, the vast majority of older adults in the overall sample were married. The proportion of married individuals in the younger-old group reached 89.2%, significantly higher than the 72.9% in the older-old group, consistent with the demographic pattern of increased probability of spousal loss at advanced ages. Regarding the number of co-residents, the average number of people living together was two to three persons. The older-old group lived with more co-residents, indicating greater reliance on family companionship. In terms of socioeconomic characteristics, over half of the older adults rated their family economic status as fair, with the overall distribution characterized by concentration in the middle range and balance at both ends. Regarding pension entitlement, 50.2% of older adults in the overall sample had pension coverage. The proportion of older-old adults with pension entitlement was higher than that of younger-old adults, indicating slightly better pension coverage in the older-old group. Regarding health-related characteristics, self-rated health status was predominantly average, accounting for 43.9%. The younger-old group reported significantly better self-rated health, with the proportion reporting “very healthy” being more than twice that of the older-old group. Additionally, the proportion of older-old adults reporting unhealthy status was higher than that of the younger-old group, consistent with the objective pattern of declining physical health with age. In terms of perceived health change compared to the previous year, 72.1% of older adults perceived no change in their health status, 20.2% perceived deterioration, and only 7.7% perceived improvement. Overall, the older adult population held a relatively negative perception of their health changes. The older-old group had even more negative health perceptions, with a higher proportion reporting health deterioration compared to the younger-old group. Regarding body mass index (BMI), the mean BMI was 23.077, which falls within the healthy weight range for Chinese adults. The mean BMI of the younger-old group was slightly higher than that of the older-old group, with neither group exhibiting significant abnormal weight issues.

**Table 1:** Results of sample characteristics.

Variable types	Variable items	Full sample (*N* = 10562)	Younger-old adults (*N* = 6622)	Older-old adults (*N* = 3940)
Dichotomous variables	Gender			
	Male	5483 (51.9%)	3411 (51.5%)	2072 (52.6%)
	Female	5079 (48.1%)	3211 (48.5%)	1868 (47.4%)
	Pension entitlement			
	Yes	5302 (50.2%)	3152 (47.6%)	2150 (54.6%)
	No	5260 (49.8%)	3470 (52.4%)	1790 (45.4%)
	Marital status			
	Not married	1785 (16.9%)	717 (10.8%)	1068 (27.1%)
	Married	8777 (83.1%)	5905 (89.2%)	2872 (72.9%)
Ordinal categorical variables	Educational level			
	Illiterate	1901 (17.5%)	795 (12.0%)	1106 (28.1%)
	Private school/literacy class	334 (3.2%)	115 (1.7%)	219 (5.6%)
	Elementary school	3822 (36.2%)	2429 (36.7%)	1393 (35.3%)
	Middle school	2942 (27.8%)	2288 (34.6%)	654 (16.6%)
	High school/vocational school	1315 (12.5%)	814 (12.3%)	501 (12.7%)
	Associate degree	218 (2.1%)	159 (2.4%)	59 (1.5%)
	Bachelor's degree or above	30 (0.30%)	22 (0.30%)	8 (0.20%)
	Economic status			
	Good	2545 (24.1%)	1570 (23.7%)	975 (24.6%)
	Fair	5905 (55.9%)	3795 (57.3%)	2110 (53.6%)
	Poor	2112 (20.0%)	1257 (19.0%)	855 (21.8%)
	Self-rated health			
	Very healthy	1479 (14.1%)	1163 (17.6%)	316(8.1%)
	Fairly healthy	3176 (30%)	1758 (26.5%)	1418(36.0%)
	Average	4640 (43.9%)	3008 (45.4%)	1632(41.4%)
	Fairly unhealthy	848 (8.1%)	484 (7.3%)	364(9.2%)
	Very unhealthy	419 (3.9%)	209 (3.2%)	210(5.3%)
	Perceived health change			
	Better	814 (7.7%)	593 (9.0%)	221 (5.6%)
	No change	7616 (72.1%)	4848 (73.2%)	2768 (70.3%)
	Worse	2132 (20.2%)	1181 (17.8%)	951 (24.1%)
Continuous variables	Bmi	23.077	23.295	22.761
	Age	73.321	–	–
	Number of co-residents	2.557	2.524	2.613

### 2.3 Measurement methods

#### 2.3.1 Dependent variable: depression

The dependent variable in this study is depression among older adults. As a core indicator of mental health assessment, depression is typically characterized by persistent low mood, diminished interest, fatigue, and poor sleep quality, exerting significant influence on the quality of life of older adults (27). To scientifically measure the level of depression, this study employs the widely validated Center for Epidemiological Studies Depression Scale (CES-D), which was revised by Cong and Silverstein and has demonstrated good reliability and validity in numerous studies on the psychology of aging (28). Specifically, the CES-D scale assesses respondents' emotional experiences over the past week through six items: (1) feeling depressed; (2) feeling lonely; (3) feeling useless; (4) poor sleep; (5) poor appetite; and (6) feeling that life is not going well. Each item offers three frequency options: 1 = rarely or none of the time, 2 = some or a little of the time, 3 = most or all of the time. Higher scores indicate a higher frequency of the corresponding depressive symptom and poorer mental health status. To further verify the applicability of this scale within the study sample, reliability and validity tests were conducted. The results show that the CES-D scale has a Cronbach's α coefficient of 0.892 and a Kaiser-Meyer-Olkin (KMO) value of 0.860 (*P* < 0.001), indicating high internal consistency and structural validity within this dataset, thus confirming its suitability for measuring depression levels among Chinese older adults.

#### 2.3.2 Independent variable: community environment

This study employs a subjective evaluation approach to assess community environment, a method that has become a mainstream evaluation paradigm in recent years within the fields of architectural planning and environmental studies (29, 30). Subjective evaluation not only directly reflects individuals' genuine experiences and affective perceptions within specific environments but also effectively captures the heterogeneity in environmental perception among demographic groups with different characteristics, such as gender, age, occupation, and health status. This provides an empirical basis for refined policy design and environmental interventions, enhancing the ecological validity and policy relevance of the data.

Specifically, the measurement of community environment in this study encompasses seven dimensions: road traffic, activity spaces, safety, environmental sanitation, lighting facilities, accessibility features, and management services. Each dimension is evaluated using a five-point Likert scale, with options ranging from “very dissatisfied” to “very satisfied”. A higher total score indicates a better perceived quality of the community environment by the respondent. To test the reliability and structural validity of this measurement instrument, reliability and validity analyses were conducted on the seven environmental indicators. The results show that the community environment scale has a Cronbach's α coefficient of 0.824 and a KMO value of 0.880 (*P* < 0.001), indicating high internal consistency and good structural validity within the context of this study, thus confirming its suitability for assessing older adults' subjective perceptions of their community environment.

#### 2.3.3 Mediator variables: aging anxiety and social adaptation stress

The first mediator variable introduced in this study is aging anxiety. This variable is measured using the Aging Anxiety Scale adopted in the CLASS database, which was originally developed by Lasher et al. and has been applied and validated in several studies on aging (31, 32). The scale consists of four items, reflecting respectively: an individual's perception of the speed of aging, the cognition of aging as a process of loss, the experience of social difficulties in old age, and feelings of rejection due to age. Each item is rated on a five-point Likert scale (1 = strongly disagree, 5 = strongly agree), with a higher total score indicating a higher level of aging anxiety. In the study sample, this scale demonstrated a Cronbach's α coefficient of 0.775 and a KMO value of 0.787 (*P* < 0.001), indicating acceptable internal consistency and good structural validity.

The second mediator variable used in this study is social adaptation stress. This variable is measured using the social adaptation scale from the CLASS database, which originates from the work of Bosc et al. and has been widely used in subsequent related research (33, 34). The scale contains four items, primarily assessing the degree of difficulty an individual experiences in adapting to the pace of social change, emerging ideas, new policies, and their impact on older adults. It employs a five-point Likert scale (1 = strongly disagree, 5 = strongly agree). A higher total score indicates a lower level of social adaptation, meaning greater difficulty in adapting to social changes. In the study sample, this scale demonstrated a Cronbach's α coefficient of 0.875 and a KMO value of 0.835 (*P* < 0.001), showing high reliability and good validity.

#### 2.3.4 Control variables

To rigorously examine the effect of community environment on depression among older adults while mitigating potential confounding bias, enhancing model robustness, and strengthening internal validity, this study followed the methodological recommendations of Bernerth and Aguinis (2016) regarding covariate selection (35). Guided by the social determinants of health framework and social integration theory, and grounded in empirical evidence, we identified and included a set of covariates with clear theoretical and empirical relevance to the core constructs. These covariates encompass four major dimensions: first, sociodemographic characteristics–gender (1 = male, 2 = female), age (continuous), and educational attainment (an ordinal scale ranging from 1 = illiterate to 7 = bachelor's degree or above); second, socioeconomic status–self-rated economic condition (1 = relatively good, 2 = average, 3 = relatively poor) and pension coverage (1 = yes, 2 = no); third, family support networks–marital status (0 = not married, 1 = married) and number of co-residing household members (ranging from 1 to 10); and fourth, health status–self-rated health (a five-point scale from 1 = very healthy to 5 = very unhealthy), perceived health change compared to the previous year (1 = improved, 2 = unchanged, 3 = worsened), and body mass index (BMI, calculated from self-reported height and weight). In the model analysis, age, number of co-residents, and BMI were treated as continuous variables, while all other control variables were treated as either dichotomous or ordinal categorical variables. All variables were measured using standardized items derived from the CLASS questionnaire. The inclusion of these covariates serves a dual purpose: it simultaneously adjusts for potential confounding arising from sociodemographic profiles, social support levels, physical health conditions, and economic resources, while avoiding the indiscriminate inclusion of extraneous variables that might compromise statistical power. This theoretically informed and empirically anchored approach enables a more precise estimation of the net association between community environment and depressive symptoms in later life.

### 2.4 Research design

This study employs structural equation modeling (SEM) to examine the complex associations linking community environment, aging anxiety, social adaptation stress, and depression among older adults. The choice of SEM is grounded in several methodological advantages. First, through confirmatory factor analysis, the model integrates multidimensional latent variables with their observed indicators, separating measurement error while more precisely capturing the essential features of abstract constructs, thereby enhancing both reliability and validity. Second, in models incorporating mediator variables, the relationship between independent and dependent variables can be partitioned into total, direct, and indirect components. SEM permits the simultaneous estimation of these effects across multiple variables, making it possible to assess the direct association between community environment and depression, systematically test the sequential mediating pathways involving aging anxiety and social adaptation stress, and further incorporate moderation analysis–all within a unified framework that comprehensively captures the network of relationships among constructs. Third, the approach provides a range of fit indices for evaluating the overall alignment between the theoretical model and observed data, and allows for nested model comparisons to identify the most plausible explanatory structure, thereby bypassing constraints inherent in traditional regression methods (36). All analyses were conducted using Mplus 8.0, with the weighted least squares mean and variance-adjusted (WLSMV) estimator employed to accommodate ordinal categorical variables.

Prior to model estimation, a series of data suitability checks were performed. First, for each observed variable, independent samples *t*-tests compared high and low scoring groups defined by the 27th and 73rd percentiles. Results indicated significant differences (*P* < 0.001) for all variables, confirming adequate discriminant validity (37). Next, the measurement properties of the four latent variables were evaluated using confirmatory factor analysis. Composite reliability values ranged from 0.734 to 0.833, all exceeding the recommended threshold of 0.7. Standardized factor loadings for observed indicators were consistently greater than or near 0.6, and the majority of squared multiple correlations exceeded 0.36, suggesting satisfactory reliability and convergent validity for all measurement models (38). Final model fit indices are presented in Table 2; all values for the full sample model met established benchmarks, indicating good alignment between the theoretical framework and empirical data. Additionally, to account for the clustering inherent in the multistage stratified sampling design of the CLASS data, community was specified as the cluster variable in the model, and robust standard errors were used to correct for within-cluster correlation and ensure the accuracy of statistical inferences (39).

**Table 2:** Comparison of model fit metrics.

Model fit metrics	TLI	CFI	RMSEA	SRMR
Full sample model	0.923	0.929	0.030	0.041
Idealized standards	>.90	>.90	< .08	< 0.08

Given that older adulthood constitutes an extended and heterogeneous life stage marked by meaningful psychological differences across age groups, this study employed multigroup structural equation modeling to investigate age-specific variation in the association between community environment and depression. Younger-old adults (Group 1, *n* = 6,622) and older-old adults (Group 2, *n* = 3,940) served as the comparison groups. The multigroup approach in SEM, which enables comparison of path coefficients and latent means across groups, offers a precise and intuitive means of identifying intergroup differences. Tests for the equivalence of corresponding path coefficients yielded significant values (*P* < 0.05), indicating that the model pathways differed meaningfully between the two age groups.

## 3 Results

### 3.1 Descriptive statistics of study variables

The descriptive statistics for the study variables reveal several notable patterns (Table 3). For perceived community environment, the observed indicators yielded mean scores centered near the 4-point threshold, suggesting that respondents' evaluations of environmental quality tended toward the moderately favorable range. Notably, assessments of accessibility features received the lowest ratings, pointing to a discernible gap in environmental modifications appropriate for the needs of older age groups for vulnerable populations within current Chinese community settings. In terms of depressive symptomatology among older adults, all corresponding observed variables registered mean scores approximately at the 2-point level. This pattern indicates a notable prevalence of depressive symptoms within the sampled population, with feelings of loneliness surfacing as the most pronounced symptom. Moreover, depressive symptoms were markedly more pronounced among older-old adults compared to their younger-old counterparts, reflecting a heavier mental health burden carried by the advanced age cohort. Scores for aging anxiety approached a mean of 4 points across its observed indicators, revealing a considerable level of concern associated with the aging process–a pattern particularly accentuated among the older-old group. Similarly, observed indicators of social adaptation stress consistently averaged above 3 points, pointing to a tangible sense of disconnection from the broader society. Taken together, these descriptive patterns underscore that the older subset of adults in later life constitute a more vulnerable subgroup, with elevated levels of depressive symptoms, anxiety related to the aging process, and social adaptation stress relative to the younger subset of this population. These findings reinforce the imperative of prioritizing the health and social integration needs of the advanced age population.

**Table 3:** Variable descriptive statistics.

Variable names	Observed variables	Variable items	Mean scores		
			Mean (All)	Mean (Younger-old adults)	Mean (Older-old adults)
Depression	DE1	During the past week, have you felt sad?	1.816	1.787	1.863
Community environment and depression	DE2	During the past week, have you felt lonely?	1.745	1.700	1.822
Community environment and depression	DE3	During the past week, do you feel useless?	1.940	1.862	2.070
Community environment and depression	DE4	During the past week, did you feel you had nothing to do?	1.933	1.862	2.052
Community environment and depression	DE5	During the past week, did you have a poor appetite?	1.816	1.782	1.873
Community environment and depression	DE6	During the past week, did you have trouble sleeping?	1.928	1.884	2.003
Community environment	CE1	Satisfaction with road traffic conditions in the community.	4.037	4.045	4.023
	CE2	Satisfaction with fitness/activity venues in the community.	3.784	3.783	3.784
	CE3	Satisfaction with the safety and security environment in the community.	3.966	3.983	3.938
	CE4	Satisfaction with environmental sanitation in the community.	3.896	3.894	3.899
	CE5	Satisfaction with road/street lighting in the community.	3.838	3.854	3.811
	CE6	Satisfaction with accessibility features in the community.	3.657	3.664	3.646
	CE7	Satisfaction with the work and services of the neighborhood/village committee.	3.886	3.885	3.889
Aging anxiety	AA1	I feel I am aging rapidly.	3.701	3.587	3.894
	AA2	In my view, aging is a continuous process of loss.	3.544	3.556	3.525
	AA3	As I age, I find it harder to make new friends.	3.653	3.588	3.764
	AA4	Because of my age, I feel excluded or left out.	3.531	3.472	3.631
Social adaptation stress	SAS1	Social changes are too rapid; I find it hard to adapt to these changes.	3.284	3.266	3.295
	SAS2	Nowadays, more and more viewpoints are difficult for me to accept.	3.240	3.236	3.243
	SAS3	Nowadays, more and more new social policies are difficult for me to accept.	3.180	3.171	3.185
	SAS4	Current social changes are increasingly unfavorable for older adults.	3.186	3.174	3.193

### 3.2 Analysis of full sample model fit results

**Figure 1:**
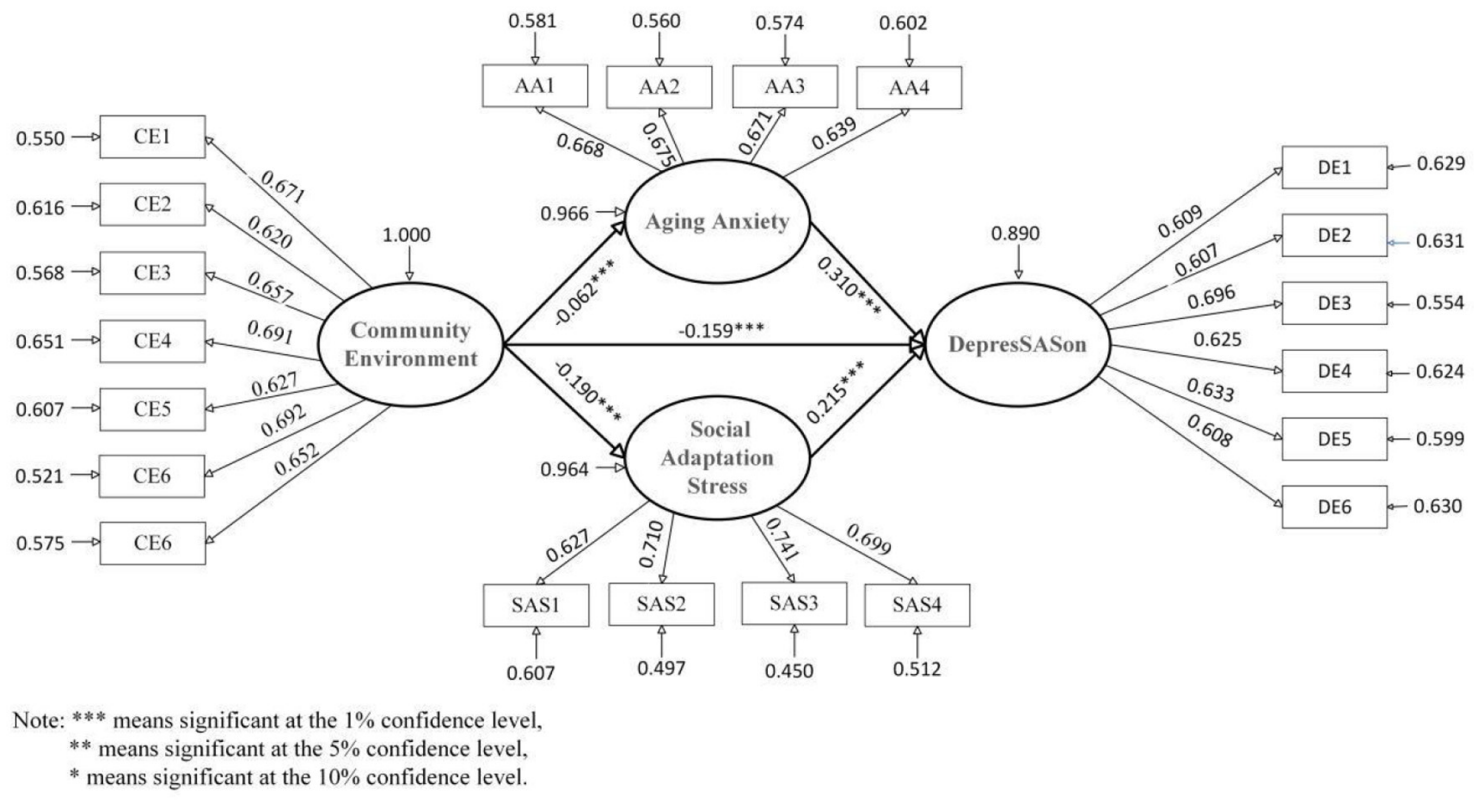
Standardized coefficients for full sample model. Note: To enhance the clarity of the visual representation, the associations of control variables on depression were simplified in the model diagram.

After adjusting for demographic and socioeconomic characteristics, the structural equation model revealed a significant negative association between community environment and depression among older adults. Examining the decomposition of effects, the total association (standardized coefficient = −0.219, unstandardized coefficient = −0.188, *P* < 0.001), direct association (standardized coefficient = −0.159, unstandardized coefficient = −0.136, *P* < 0.001), and indirect association (standardized coefficient = −0.060, unstandardized coefficient = −0.051, *P* < 0.001) all reached statistical significance, with the direct component exceeding the indirect component in magnitude. Concretely, a one-unit improvement in community environment corresponded to a direct decrease in depression scores of 0.136 points on average, while simultaneously linking to an indirect decrease of 0.051 points through mediating pathways—the latter accounting for 25.8% of the total association. This pattern suggests that community environment is not only directly related to lower levels of depression in older adults but also operates partially through indirect channels. Tables 4, 5, and Figure 1 present the complete results of the final model.

**Table 4:** Standardized path results of the association between community environment and depression of older adults.

Variables			Mediator variables		Dependent variable:Depression		
			Aging anxiety	Social adaptation stress	Total effect	Direct effect	Indirect effect
Independent variable	Community environment		−0.062^***^	−0.190^***^	−0.219^***^	−0.159^***^	−0.060^***^
Mediator variables	Aging anxiety					0.310^***^	
	Social adaptation **stress**					0.215^***^	
Mediation paths	Community environment →	→ Aging anxiety → depression					−0.019^***^
		→ Social adaptation **stress** → depression					−0.041^***^
Control variables	Gender					−0.036^***^	
	Age					−0.048^***^	
	Educational level					0.042^***^	
	Marital Status					0.073^***^	
	Number of cohabitants					−0.047^***^	
	Self-rated health					−0.188^***^	
	Perceived health change					−0.003	
	BMI					0.018	
	Economic status					−0.004	
	Pension entitlement					0.049^***^	

**Table 5:** Non-standardized path results of the association between community environment and depression of older adults.

Variables			Mediator variables		Dependent variable:Depression		
			Aging anxiety	Social adaptation stress	Total effect	Direct effect	Indirect effect
Independent variable	Community environment		−0.072^***^	−0.257^***^	−0.188^***^	−0.136^***^	−0.051^***^
Mediator variables	Aging anxiety					0.226^***^	
	Social adaptation **stress**					0.136^***^	
Mediation paths	Community environment →	→ Aging anxiety → depression					−0.016^***^
		→ Social adaptation **stress** → depression					−0.035^***^

Further decomposition of the two parallel mediating pathways revealed significant indirect associations for both the “community environment → aging anxiety → depression” and “community environment → social adaptation stress → depression” sequences. For the aging anxiety pathway, the mediating effect was (standardized coefficient = −0.019, unstandardized coefficient = −0.016, *P* < 0.001), indicating that each one unit improvement in community environment was linked to a significant 0.072 point reduction in aging anxiety (*P* < 0.001), which in turn corresponded to an average 0.016 point decrease in depression scores. For the social adaptation stress pathway, the mediating effect was (standardized coefficient = −0.041, unstandardized coefficient = −0.035, *P* < 0.001), reflecting that each one unit enhancement in community environment was associated with a substantial 0.275 point reduction in social adaptation stress (*P* < 0.001), subsequently relating to an average 0.035 point decrease in depression. These findings demonstrate that both aging anxiety and social adaptation stress function as partial mediators in the relationship between community environment and depression among older adults. Of particular note, the mediating effect of social adaptation stress was significantly larger than that of aging anxiety, suggesting that within the mental health pathways linked to community environment, the channel operating through reduced social adaptation stress may carry greater practical significance for intervention efforts aimed at alleviating depression in later life.

### 3.3 Comparison of model paths across different age groups

**Figure 2:**
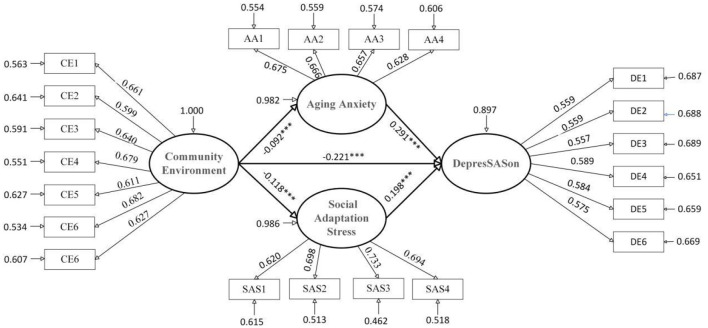
Standardization coefficients for younger-old adults model.

**Figure 3:**
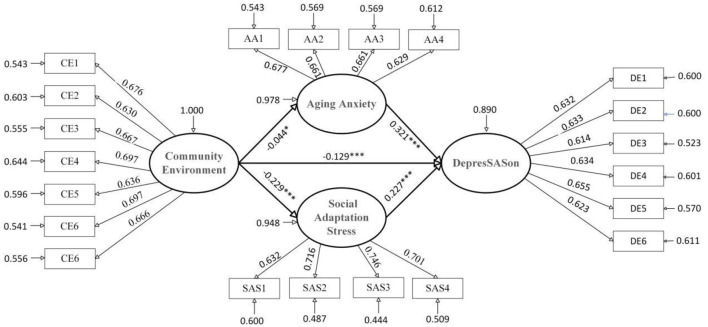
Standardization coefficients for older-old adults model.

Tables 6, 7, along with Figures 2 and 3, present the model fit comparisons across age-stratified samples of older adults. After accounting for relevant covariates, a significant negative association emerged between community environment and depression among the younger-old group. Examining the decomposition of effects, the total association (standardized coefficient = −0.195, unstandardized coefficient = −0.168), direct association (standardized coefficient = −0.129, unstandardized coefficient = −0.111), and indirect association (standardized coefficient = −0.066, unstandardized coefficient = −0.057) all reached statistical significance, with the direct component exceeding the indirect component in magnitude. This pattern suggests that the link between community environment and lower depression scores among the younger-old operates primarily through direct channels, with only a modest portion channeled indirectly via the proposed mediators. Specifically, a one unit improvement in community environment corresponded to a direct decrease in depression scores of 0.111 points on average, while simultaneously relating to an indirect decrease of 0.057 points through mediating pathways.

**Table 6:** Standardized path analysis results of the association between community environment and depression among older adults across different age groups.

Variables			Mediator variables		Dependent variable:Depression		
			Aging anxiety	Social adaptation stress	Total effect	Direct effect	Indirect effect
A: Younger-old adults							
Independent variable	Community environment		−0.044^*^	−0.229^***^	−0.195^***^	−0.129^***^	−0.066^***^
Mediator variables	Aging anxiety					0.321^***^	
	Social adaptation **stress**					0.227^***^	
Mediation paths	Community environment →	→ aging anxiety → depression					−0.014^*^
		→ social adaptation **stress** → depression					−0.052^***^
B: older-old adults							
Independent variable	Community environment		−0.092^***^	−0.118^***^	−0.271^***^	−0.221^***^	−0.050^***^
Mediator variables	Aging anxiety					0.291^***^	
	Social adaptation stress					0.198^***^	
Mediation paths	Community environment →	→ aging anxiety → depression					−0.027^***^
		→ social adaptation stress → depression					−0.023^***^

**Table 7:** Non-standardized path analysis results of the association between community environment and depression among older adults across different age groups.

Variables			Mediator variables		Dependent variable:Depression		
			Aging anxiety	Social adaptation stress	Total effect	Direct effect	Indirect effect
A: Younger-old adults							
Independent variable	Community environment		−0.051^*^	−0.307^***^	−0.168^***^	−0.111^***^	−0.057^***^
Mediator variables	Aging anxiety					0.236^***^	
	Social adaptation **stress**					0.145^***^	
Mediation paths	Community environment →	→ aging anxiety → depression					−0.012^*^
		→ social adaptation **stress** → depression					−0.045^***^
B: older-old adults							
Independent variable	Community environment		−0.113^***^	−0.163^***^	−0.225^***^	−0.183^***^	−0.042^***^
Mediator variables	Aging anxiety					0.196^***^	
	Social adaptation stress					0.119^***^	
Mediation paths	Community environment →	→ aging anxiety → depression					−0.022^***^
		→ social adaptation stress → depression					−0.019^***^

Further decomposition of the two parallel mediating pathways revealed significant indirect associations for both the “community environment → aging anxiety → depression” and “community environment → social adaptation stress → depression” sequences. For the aging anxiety pathway, the mediating effect was (standardized coefficient = −0.014, unstandardized coefficient = −0.012), indicating that each one unit improvement in community environment was linked to an average 0.012 point reduction in depression scores through its relation to lower aging anxiety. For the social adaptation stress pathway, the mediating effect was (standardized coefficient = −0.052, unstandardized coefficient = −0.045), reflecting that each one unit enhancement in community environment corresponded to an average 0.045 point decrease in depression through its association with reduced social adaptation stress. Together, these findings demonstrate that both aging anxiety and social adaptation stress function as partial mediators in the relationship between community environment and depression among the younger-old, jointly constituting parallel pathways through which community environment relates to mental health in this subgroup.

Turning next to the older-old group, after accounting for relevant covariates, a significant negative association again emerged between community environment and depression. The decomposition of effects revealed that the total association (standardized coefficient = −0.271, unstandardized coefficient = −0.225), direct association (standardized coefficient = −0.221, unstandardized coefficient = −0.183), and indirect association (standardized coefficient = −0.050, unstandardized coefficient = −0.042) all reached statistical significance, with the direct component substantially exceeding the indirect component. This pattern indicates that the link between community environment and lower depression scores among the older-old operates predominantly through direct channels, with a smaller proportion channeled indirectly: a one unit improvement in community environment corresponded to a direct decrease in depression scores of 0.183 points on average, while simultaneously relating to an indirect decrease of 0.042 points through mediating pathways—the latter accounting for 18.5% of the total association.

Further decomposition of the two parallel mediating pathways again revealed significant indirect associations for both sequences. For the aging anxiety pathway, the mediating effect was (standardized coefficient = −0.027, unstandardized coefficient = −0.022), indicating that each one unit improvement in community environment was linked to an average 0.022 point reduction in depression scores through its relation to lower aging anxiety. For the social adaptation stress pathway, the mediating effect was (standardized coefficient = −0.023, unstandardized coefficient = −0.019), reflecting that each one unit enhancement in community environment corresponded to an average 0.019 point decrease in depression through its association with reduced social adaptation stress. Together, these findings demonstrate that both aging anxiety and social adaptation stress function as partial mediators in the relationship between community environment and depression among the older-old, jointly constituting parallel pathways through which community environment relates to mental health in this subgroup, with the two pathways contributing to a comparable degree.

In summary, the comparative analysis based on age groups reveals a significant differential pattern: the negative association between community environment and depression is stronger for the older-old group than for the younger-old group. This finding suggests that the depressive status of older-old adults exhibits higher sensitivity to environmental suitability compared to their younger-old counterparts. Further mediation tests confirm that both social adaptation stress and aging anxiety exert significant mediating effects in both groups. Notably, however, the mediating role of aging anxiety is more pronounced in the older-old group, whereas the mediating role of social adaptation stress is distinctly more significant in the younger-old group.

### 3.4 Robustness check using multiple imputation

To evaluate whether the application of listwise deletion may have introduced bias in parameter estimates through the exclusion of incomplete cases, multiple imputation was employed to handle missing data in the original sample (*N* = 11,670). Mediation models were re-estimated across 20 imputed datasets, and the pooled estimates were compared with those derived from the primary analysis using listwise deletion (*N* = 10,562).

The results demonstrated a high degree of consistency in key parameter estimates between the two missing data handling approaches. Specifically, in the multiple imputation analysis, the pooled estimate for the total association between community environment and depression among older adults yielded an unstandardized coefficient of *B* = −0.186 (95% CI:−0.212 to−0.160), the direct association was B = −0.135 (95% CI:−0.159 to−0.111), and the indirect association was B = −0.051 (95% CI:−0.062 to−0.040)–all closely overlapping with the estimates obtained through listwise deletion. The path coefficients exhibited substantial concordance, and the significance levels (*P* < 0.001) remained unchanged across all estimates. These findings indicate that the parameter estimates in this study are not sensitive to the choice of missing data handling method. Although the original data contained 9.5% missingness, the conclusions drawn from listwise deletion and multiple imputation were entirely aligned, thereby confirming the robustness of the results and mitigating concerns regarding estimation bias stemming from the exclusion of incomplete cases.

## 4 Discussion

Drawing on a sample of 10,562 older adults from the CLASS 2023, this study constructed an integrated conceptual framework positioning aging anxiety and social adaptation stress as mediating variables linking community environment to depression. Through structural equation modeling, it elucidated the nuanced associations between community environment and the mental health of older adults, along with their variations across different age groups.

The analysis first revealed that older-old adults constitute a comprehensively vulnerable subgroup, exhibiting more pronounced levels of depression, aging anxiety, and social adaptation stress compared to their younger-old counterparts. Consistent with prior gerontological research, this pattern underscores the imperative of prioritizing the health needs of the advanced age population (40). With respect to community environment, older adults reported the highest satisfaction with road traffic conditions while expressing the greatest discontent with age-friendly environmental designs. This finding closely mirrors real conditions and carries important implications: community planning must extend beyond basic infrastructure to explicitly address the needs of vulnerable groups. Age-adapted environments are essential for supporting the daily activities and overall health of older adults.

Second, this study revealed a significant negative association between community environmental quality and depressive symptoms among older adults. This finding aligns closely with the World Health Organization's conceptual frameworks of “Healthy Cities” and “Age-friendly Communities,” which posit that the community–as the fundamental spatial unit of daily life–constitutes a key social determinant of health through its design and management. A supportive community environment, characterized by ample green and public spaces, safe and convenient walkable infrastructure, is associated with better mental health outcomes. This result corroborates a growing body of international evidence. For instance, Astell-Burt et al. (2021) (7) documented an association between neighborhood green space accessibility and lower depression risk. Similarly, research by Kelly et al. (2018) (8) indicated that older adults residing in highly walkable neighborhoods reported fewer depressive symptoms, an association partly mediated by higher neighborhood social cohesion. The present study validates a similar pattern across 30 Chinese provinces, reinforcing the importance of improving community environments as a fundamental public health strategy that moves beyond treatment at the individual level to actively shape the upstream determinants of health.

Further mediation analysis revealed that the association between community environment and depression is not entirely direct; rather, it is partially channeled through two distinct pathways: the reduction of aging anxiety and the alleviation of social adaptation stress. This finding represents an advance in environmenal health research by illuminating the underlying mechanisms at play. First, the mediating role of aging anxiety suggests that a safe, convenient, and inclusive community environment may mitigate negative emotions arising from age-related physical decline. When environmental features such as level walkways, ample seating, and clear signage compensate for functional limitations, older adults‘ sense of self-efficacy is preserved, which in turn is associated with fewer depressive symptoms (22). Second, and of particular note, social adaptation stress exhibited a stronger mediating effect. This suggests that, within the association chain linking community environment to depression, the environment operates primarily by improving older adults' state of social adaptation and reducing their adaptive stress. A community characterized by a high level of age friendliness and a supportive atmosphere can ease the adaptive burden older adults face when navigating environmental changes, mitigate psychological tension, and thereby contribute to lower levels of depressive symptoms (10, 11). Through parallel mediation analysis, this study offers a novel contribution by comparing, within a single model, the relative roles of aging anxiety and social adaptation stress in the environment health pathway. The stronger indirect effect observed for social adaptation stress points to a crucial insight: the mental health benefits associated with community environments may depend not merely on their function as “physical infrastructure” but, more critically, on their role as social infrastructure.

More importantly, the study revealed significant age-group heterogeneity in both the associations between community environment and depression among older adults and the underlying mediating mechanisms. Specifically, the direct association between community environment and depression was stronger for older-old adults, and this link was channeled to a greater extent through the pathway involving the alleviation of aging anxiety. For younger-old adults, by contrast, the indirect association was relatively more prominent and operated primarily through the pathway of reduced social adaptation stress. This finding shifts the research perspective from viewing “older adults” as a homogeneous population to recognizing the highly heterogeneous subgroups within it, offering valuable insights into the life stage dynamics of environmental influences. For older-old adults, the community environment exhibits a stronger direct protective association, with a mediating pathway centered on aging anxiety. This pattern likely reflects the reality that, as individuals advance into advanced age, they experience greater physical decline, a contraction in their activity range, and a sharply increased dependence on their immediate living environment (24, 25). At this stage, a supportive and accessible community environment becomes directly critical for maintaining basic daily activities and preserving a sense of autonomy, thereby most effectively countering the aging anxiety exacerbated by the risk of physical disability. While previous studies have often noted the importance of the community environment for the overall health of older-old adults, they have seldom isolated the key proximal psychological mechanism of “aging anxiety” (26). In stark contrast, a markedly different pattern emerged for younger-old adults: a substantially larger proportion of the indirect association was channeled through the “social” pathway of fostering adaptive capacity. This divergence likely stems from the unique life stage of adults in the younger bracket of later life, who are undergoing the critical transition from workforce participation to retirement, a period marked by profound shifts in social roles. For this cohort, one of the central developmental tasks involves reconstructing social identities outside of paid work and adapting to the social environment and daily rhythm of life after retirement (41). Consequently, the community environment, as the primary arena for their daily activities, assumes heightened significance. Whether the community provides adequate adaptive support–such as services tailored to their needs, an inclusive neighborhood atmosphere, and activities designed to facilitate social reengagement–becomes crucial in mitigating the social adaptation stress that may arise from retirement and the accompanying loss of professional roles.

This finding of age group heterogeneity represents a central theoretical contribution of the present study. It moves beyond prior research paradigms that often treat the associations of community environments as static or uniform, instead revealing a dynamic portrait in which the core mechanisms of association shift across the life course–transitioning from a focus on rebuilding social connections to one centered on preserving autonomy and a sense of security. This pattern strongly suggests that understanding the relationship between community environment and the health of older adults requires adopting a developmental perspective sensitive to different life stages.

Based on the observed negative association between community environment and depression in older adults, the dual mediating roles of aging anxiety and social adaptation stress, and the moderating effect of age, we propose the following three recommendations for community design and management, all of which are broadly applicable and responsive to the specific needs of subgroups across different ages. First, we recommend implementing a dual transformation strategy across all communities to strengthen the overall mental health protective effect. In response to older adults' consistently low ratings of accessible facilities, priority should be given to upgrading age-friendly infrastructure such as corridors and ramps, while improving basic environmental conditions including lighting and public safety. These enhancements are intended to reduce physical barriers and safety concerns that hinder outdoor activity. Concurrently, community activity spaces should be leveraged to foster social engagement opportunities. Low threshold activities such as neighborhood markets and group morning exercises can help mitigate social adaptation stress among older adults, thereby building a foundation for mental health promotion from both physical and social dimensions. Second, with a focus on promoting mental health among the older-old, community environment optimization should specifically target the reduction of aging anxiety, thereby reinforcing the direct protective association. To this end, offering lectures on social development and health, along with dedicated digital device learning zones in community public spaces, can help alleviate the stress associated with social adaptation amid perceived cognitive decline. Such initiatives support older adults in maintaining self-efficacy and help foster a sense of engagement with contemporary societal progress. Third, for the younger-old, we recommend establishing a adaptive support ecosystem specifically designed to alleviate social adaptation stress, thereby activating and enhancing their adaptive capacity. Centered on facilitating social participation and rebuilding social roles, this approach involves creating digital functional zones within community spaces and developing learning platforms for older adults. Providing opportunities for younger-old individuals to engage with contemporary societal developments can support them in navigating the challenges of social role transitions following retirement, thus helping to alleviate social adaptation stress and facilitate the reconstruction of worth.

The present study also has several limitations that point to directions for future research. First, as the data are derived from the cross-sectional CLASS 2023 survey, causal inference remains limited despite the valid testing of associations and mediating pathways. Future studies are advised to employ longitudinal data to further elucidate the dynamics and directionality of these relationships. Second, the measurement of community environment in this study relied primarily on participants' subjective evaluations, without incorporating objective indicators. This approach not only renders the findings susceptible to common method bias but also limits the comprehensive reflection of actual community conditions, potentially introducing measurement bias into the observed associations between community environment and the study variables. An important direction for future research involves integrating the CLASS dataset with objective community indicators, such as green space coverage, number of public facilities, and road density obtained through Geographic Information Systems. By synthesizing subjective perceptions with objective metrics, future studies can enhance the robustness and comprehensiveness of community environment measurement, thereby yielding more precise insights into the mechanisms linking community environment to late life depression. Finally, while utilizing the most recent nationally representative data on older adults available at the time, the data were collected in 2023. Given the accelerating pace of population aging and the rapid transformation of urban and rural environments in China, the timeliness of the findings may be constrained. Subsequent research based on updated data is necessary to continuously track the evolving relationship between community environment and depression among older adults.

## 5 Conclusion

The results reveal a considerable prevalence of depressive symptoms among Chinese older adults, alongside notable levels of aging anxiety and social adaptation stress, challenges that are especially acute among adults in the older bracket of later life. Guided by a theoretical framework positioning social adaptation stress and aging anxiety as parallel mediators, the empirical analysis yields three key findings. First, community environment is significantly and negatively associated with depression. Second, both social adaptation stress and aging anxiety partially mediate this relationship, with the indirect effect of social adaptation stress being more pronounced. Third, and most importantly, age moderates these associations. Among the older-old, the community environment–depression link is stronger and operates more prominently through the alleviation of aging anxiety. Among the younger-old, by contrast, the indirect pathway is more robust and is channeled primarily through reduced social adaptation stress. These findings advance social adaptation theory by elucidating the mechanisms through which community environment shapes mental health in later life, while also offering empirical guidance for age friendly community initiatives. Future environmental interventions should attend to both social connectedness and psychological adaptation, and should be tailored to the specific needs of different age groups. Such targeted approaches hold promise for systematically enhancing mental health among older adults and advancing the broader goals of healthy aging.

## Data Availability

The raw data supporting the conclusions of this article will be made available by the authors, without undue reservation.
